# Information perception, wishes, and satisfaction in ambulatory cancer patients under active treatment: patient-reported outcomes with QLQ-INFO25

**DOI:** 10.3332/ecancer.2014.425

**Published:** 2014-05-02

**Authors:** Ana Catarina Pinto, Fernando Ferreira-Santos, Lissandra Dal Lago, Evandro de Azambuja, Francisco Luís Pimentel, Martine Piccart-Gebhart, Darius Razavi

**Affiliations:** 1 Medicine Department, Medical Oncology Unit, Institut Jules Bordet, Université Libre de Bruxelles, Boulevard de Waterloo, 121 (7^th^ Floor), 1000 Brussels, Belgium; 2 Br.E.A.S.T. Data Centre, Institut Jules Bordet, Brussels 1000, Belgium; 3 Laboratory of Neuropsychophysiology, Faculty of Psychology and Education Sciences, University of Porto, Porto 4200-135, Portugal; 4 Developmental Cognitive Neuroscience Unit, UCL Institute of Child Health, London WC1N 1EH, UK; 5 Health Sciences, University of Aveiro, Aveiro 3810-193, Portugal; 6 Psychosomatic and Psycho-Oncology Research Unit, Université Libre de Bruxelles, Brussels 1050, Belgium; 7 Psycho-Oncology Clinic, Institut Jules Bordet, Université Libre de Bruxelles, Brussels 1000, Belgium

**Keywords:** ambulatory care facility, cancer, information, physician–patient relations, quality of life, questionnaires

## Abstract

**Background:**

Information is vital to cancer patients. Physician–patient communication in oncology presents specific challenges. The aim of this study was to evaluate self-reported information of cancer patients in ambulatory care at a comprehensive cancer centre and examine its possible association with patients’ demographic and clinical characteristics.

**Patients and methods:**

This study included adult patients with solid tumours undergoing chemotherapy at the Institute Jules Bordet’s Day Hospital over a ten-day period. EORTC QLQ-C30 and QLQ-INFO25 questionnaires were administered. Demographic and clinical data were collected. Descriptive and inferential statistics were used.

**Results:**

101 (99%) fully completed the questionnaires. They were mostly Belgian (74.3%), female (78.2%), with a mean age of 56.9 ± 12.8 years. The most frequent tumour was breast cancer (58.4%). Patients were well-informed about the disease and treatments, but presented unmet information domains. The Jules Bordet patients desired more information on treatment side effects, long-term outcome, nutrition, and recurrence symptoms. Patients on clinical trials reported having received less information about their disease and less written information than patients outside clinical trials. Higher information levels were associated with higher quality of life (QoL) scores and higher patient satisfaction.

**Conclusion:**

Patients were satisfied with the information they received and this correlated with higher QoL, but they still expressed unmet information wishes. Additional studies are required to investigate the quality of the information received by patients enrolled in clinical trials.

## Introduction

Patients reporting good communication with their doctors are more likely to be satisfied with their care, share pertinent information for accurate diagnosis, follow advice, and adhere to treatments [1–3]. Surveys consistently show that patients want better communication with their doctors [2]. Moreover, they show that communication problems correlate positively with patients’ distress and anxiety, causing anticipatory nausea and vomiting during chemotherapy, criticism about information received during hospital visits, and even low rates of recruitment into clinical trials [2, 3]. These circumstances are also professionally and personally unrewarding for health-care staff [1, 2].

Earlier studies evaluating the delivery of information according to patients’ needs recognised that giving patient-tailored information contributed to lowering their anxiety levels [4]. Such studies have also shown that doctors’ decision-making on giving information is based on subjective criteria with a strong cultural influence; it correlates poorly with paired patients’ assessments and causes the physician to attain a conservative attitude [4–6]. Reasons may include both personality and attitudinal characteristics of patients and their health carers. Issues at the level of the cancer care delivery systems also constitute an impediment to good communication [5].

Doctors rate poorly in studies identifying patient misunderstanding, typically underestimating the proportion of patients misreporting information [7]. These results are in agreement with the body of research demonstrating that most clinicians cannot accurately detect patient emotional states [7, 8]. Evidence shows that few doctors recognise the different preferences that their patients have for both type and amount of information. The desire for more information is sometimes confused with a presumed wish to participate in clinical decision-making. For example, in women with breast cancer, research has shown that the majority prefers a relatively passive role in decision-making but require, nevertheless, a large amount of information regarding treatment options [9].

To better understand how cancer patients perceive the information they receive from health-care professionals and making use of a tool devised with this specific purpose, with the aim of delivering tailored standardised information to patients in our centre at a later time point, we carried out a cross-sectional observational study assessing the self-reported information regarding disease, treatment, prognosis, and supportive care in patients attending the ambulatory care facility of our comprehensive cancer centre.

## Patients and methods

### Patients

Eligible patients were adults (≥18 years) with histologically proven solid tumours, receiving any line of intravenous chemotherapy at the Institute Jules Bordet’s (IJB) Day Hospital in Brussels, Belgium. They also had to be literate and fluent in either French or English. Patients with neurological impairment preventing the comprehension of the questionnaires were excluded. Ethics committee approval was obtained, as was informed consent for all patients before study enrollment.

### Questionnaires

Patients completed the EORTC QLQ-C30 questionnaire (version 3.0) and its information module EORTC QLQ-INFO25. The EORTC QLQ-C30 (v. 3.0) is a validated, widely used questionnaire, suitable for observational, experimental, and randomised clinical trials, containing five functional scales (physical, role, emotional, social, and cognitive function), three symptom scales (fatigue, pain, and nausea/vomiting), one global health status/quality of life (QoL) scale, and six single items (symptoms and financial impact) [10]. It contains 30 questions, and the response format is a four-point Likert scale (1 = not at all, 2 = a little, 3 = quite a bit, and 4 = very much) for questions 1–28. The response format for questions 29 and 30 is a seven-point scale (ranging from 1—very poor to 7—excellent). The questions are answered by circling the option the patient considers applicable to him/her [10].

Validated by Arraras *et al *[11] as a reliable measure of patients’ perceptions of received information, QLQ-INFO25 (hereupon referred to as INFO25) is a module of the EORTC QLQ-C30 that can be used in clinical practice and research to evaluate information-based interventions and programmes [11]. It contains four multi-item scales (information about the disease, medical tests, treatments, and other services) and eight single items (e.g., places of care, self-help to get well, information channels, and information satisfaction and usefulness). INFO25 also includes two open questions allowing patients to write about topics of their choice. Overall, INFO25 comprises 25 questions in a four-point Likert scale response format (1 = not at all, 2 = a little, 3 = quite a bit, and 4 = very much), except for the dichotomous (yes/no) questions 51 and 52 and 54 and 55 (for questionnaire specimens please visit http://groups.eortc.be/qol/eortc-qlq-c30 and http://groups.eortc.be/qol/why-do-we-need-modules).

Demographic data (age, gender, marital status, level of education, profession, and place of birth) were collected. Clinical data (primary tumour site, extent of disease [limited/disseminated], World Health Organisation performance status [ECOG], and treatment line received) were also collected, through medical chart review and direct questioning of patients in case of missing chart information.

### Procedures

Every one out of three eligible patients was identified from IJB’s Day Hospital treatment daily schedules and was invited to participate while in the Day Hospital setting. Participation only took place after informed consent signature.

The same person (A.C.P.) applied the questionnaires for all the patients included in this study. Questionnaires were administered on a paper-printed format, self-reported on a paper-and-pencil basis. Administration time was estimated to be 10–15 min for each of the EORTC questionnaires. The investigator could read out the questions for the patients if required, and write the answers on the form, without exerting any kind of influence towards answer choice. Patients answered the questionnaire in the presence of the investigator and in the absence of proxy (to allow for privacy and avoid interference).

### Statistical considerations

A power analysis using G*Power3 software (version 3.1.2.) was conducted to estimate the appropriate sample size (12). Following Arraras *et al*’s specifications [11], it was estimated that a sample of 94 patients would have 90% power to detect a ten-point difference in responses in a two-tailed Mann–Whitney *U* test at 0.05 significance.

To address the primary objective (participants’ self-reported information), the data collected were summarised by obtaining descriptive statistics of central tendency and variability (significance level of 0.05). Cronbach’s alpha values were computed for the INFO25 scales defined by Arraras *et al *to assess their internal consistency [11]. Descriptive statistics of scale responses for subgroups of interest were also computed, namely those defined by age, gender, birthplace, marital status, education level, profession, primary tumour site, performance status (ECOG), disease extent, and treatment line received. To address association between self-reported information and socio-demographic and clinical characteristics, eight *a priori *hypotheses (H) based on the literature [6, 13–17] were tested, using Mann–Whitney *U *tests (one or two-tailed, as appropriate):

*H1:* Women’s information level > men’s information level.

*H2:* Women’s information satisfaction < men’s information satisfaction.

*H3: *Younger patients’ information level (≤65 years) > older patients’ information level (66–84; ≥85 years).

*H4:* Information level of patients with higher education > information level of patients with lower education.

*H5:* Written information of less educated patients < written information of more educated patients.

*H6:* Wishes of patients with lower education < wishes of patients with higher education.

*H7:* Information level under curative treatment ≠ information level under palliative treatment.

*H8:* Information level on ≥ second chemotherapy line ≠ information level on first chemotherapy line.

The remaining clinical and demographic characteristics of the patients were subjected to an exploratory analysis, and comparison of item and scale responses between clinical and demographic subgroups were performed by means of Mann–Whitney *U *tests (for comparisons between two groups) or Kruskal–Wallis one-way analysis of variance (for comparisons between more than two groups). Unplanned exploratory hypotheses tests may lead to the inflation of the Type I error rate due to multiple comparisons. However, because of the potential clinical relevance of the findings, the significance level (*α*) for the exploratory analyses was kept at 0.05, and inferences were performed on uncorrected *p*-values. Bonferroni corrected results were further calculated and statistically significant comparisons that survive the correction were signaled where appropriate.

All statistical analyses were conducted using Microsoft Excel and SPSS v17.0 (SPSS Inc., Chicago, Illinois, USA).

## Results

### Demographic and clinical variables—descriptive statistics

Between 30 May and 8 June 2011, 102 patients were offered the chance to participate in the study, and all agreed to participate. 101 patients (99%) fully completed both questionnaires. One patient was too tired to complete the questionnaire and was therefore excluded from the analysis.

The patients’ demographic and clinical characteristics are presented in Table 1. Twenty-nine patients (28.7%) were participating in a clinical trial. The majority had breast cancer diagnosis (59 patients; 58.4%).

### INFO25 questionnaire—descriptive statistics

The INFO25 scales showed good internal consistency, with Cronbach’s alpha values in line with the findings of Arraras *et al *[11]. The descriptive statistics of INFO25 scales/items, internal consistency and comparison with the module’s validation study are shown in Table 2.

### INFO25 open questions (wishes about more or less information)

Patients were asked to respond to the two open questions contained in INFO25, that is, to list topics they would either like to receive *more *information about (question 53b, following affirmative response to 53a), or *less *information about (question 54b, following affirmative response to 54a). Regarding question 53b, we separated the answers dichotomously into: (1) topics already mentioned in INFO25 and (2) new topics suggested by the patients. As for question 54a, only one patient responded affirmatively, writing in 54b that she desired to receive less information ‘when her health condition deteriorated’.

Fifty-six patients answered item 53a affirmatively (three did not answer question 53b: designated as missing items). The most frequent INFO25 topics about which patients expressed their wish to receive more information were possible treatment side effects (item 40; 11 patients) and results of the medical tests already performed (item 37; nine patients) [data not shown]. The predominant new topics on which patients wanted more information were long-term outcome (eight patients), nutrition (four patients), and recurrence symptoms (four patients) [data not shown].

## Inferential statistics

Of the eight hypotheses on association between self-reported information and the socio-demographic and clinical characteristics of patients, three associations were significant while five were not. The statistically significant associations were related to age (*H3*), treatment type (*H7*), treatment lines (*H8*), the non-significant ones were linked to gender (*H1* and *H2*), and education (*H4*, *H5*, and *H6*).

Regarding age (*H3*), self-reported information was statistically significantly higher in older patients (≥66 years old) for written information (*M *= 62.1, *SD *= 49.4 versus *M *= 36.1, *SD *= 48.4; *p *= 0.02) and information on CD/tape/video (*M *= 100.0, *SD *= 0 versus *M *= 86.1, *SD *= 34.8; *p *= 0.03). In younger patients (≤65 years old) the results were significant regarding satisfaction with the information received (*M *= 67.6, *SD *= 28.0 versus *M *= 59.8, *SD *= 22.5; *p *= 0.049) and helpfulness of the overall information received (*M *= 78.2, *SD *= 22.5 versus *M* = 66.7, *SD* = 23.6; *p *= 0.01).

With reference to treatment type (*H7*), self-reported information was statistically significantly higher in patients undergoing treatment with curative intent (*M *= 60.0, *SD *= 35.1) than in patients receiving palliative treatment (*M *= 33.8, *SD *= 34.8) with respect to self-help to get well (*p *= 0.001) and also to overall helpfulness of information (*M *= 82.9, *SD *= 20.4 versus *M *= 70.7, *SD *= 23.8; *p *= 0.01). The reverse association was true with regard to the information on CD/tape/video, with higher mean scores for patients undergoing palliative treatment than those being treated with curative intent (*M* = 98.5, *SD* = 12.3 versus *M* = 74.3, *SD* = 44.3; *p* < 0.001, respectively).

Finally, regarding treatment lines (*H8*), patients who had undergone two or more chemotherapy lines presented a higher mean score for the information obtained from CD/tape/video than patients having received only one line (*M *= 100.0, *SD *= 0 versus *M *= 81.5, *SD* = 39.2; *p* = 0.002).

## Exploratory analyses

Regarding place of birth and marital status, the only statistically significant association was a higher score for satisfaction with information received by patients born outside of Belgium (*M* = 74.4, *SD* = 23.7 versus *M* = 62.2, *SD* = 27.0; *p* = 0.05).

**Figure 1:**
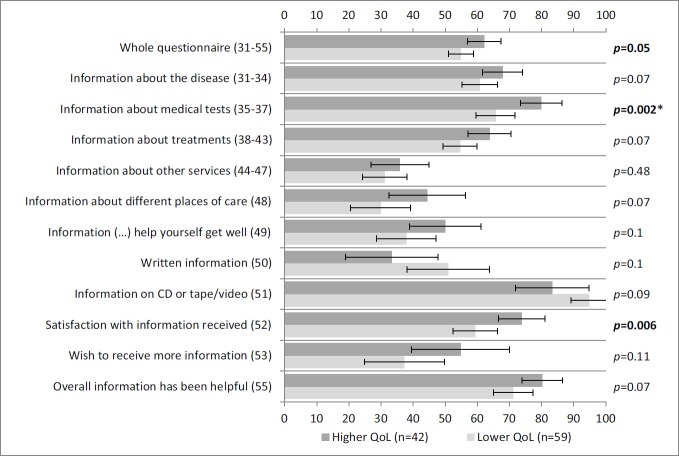
Self-reported information according to the QoL scale of QLQ-C30. A lower QoL (lowest through mean) and higher QoL (mean through highest) correspond to scores on the QoL scale of QLQ-C30. Vertical axis: INFO25 questions; horizontal axis: scores on the INFO25 questions (items range from 0 to 100). Higher scores mean a higher level of information received, higher information wishes, and higher satisfaction. Error bars represent 95% confidence intervals. *P*-values (Mann–Whitney *U* tests) appear on the right, with significant *p*-values shown in bold and comparisons that remain statistically significant after Bonferroni correction signalled with an asterisk.

Figure 1 shows the comparisons made with the Global QoL scale of the QLQ-C30 questionnaire. Self-reported information was statistically significantly higher in patients with higher QoL scores for the whole questionnaire, information about medical tests, and satisfaction with information.

**Figure 2:**
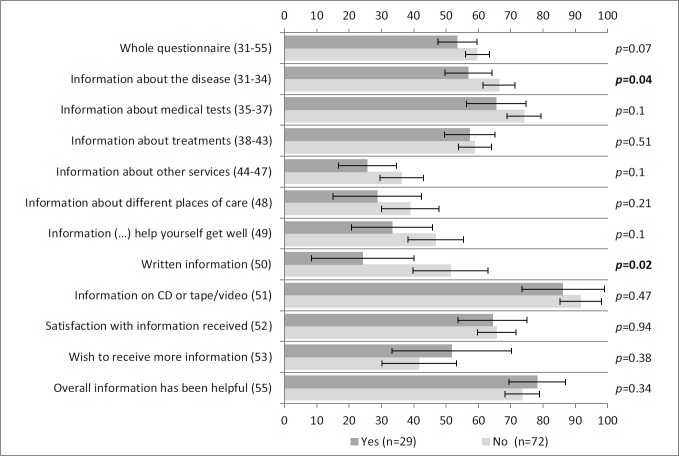
Self-reported information according to clinical variables: clinical trial participation. Vertical axis: INFO25 questions; horizontal axis: scores on the INFO25 questions (items range from 0 to 100). Higher scores mean a higher level of information received, higher information wishes, and higher satisfaction. Error bars represent 95% confidence intervals. P-values appear on the right, with significant p-values (Mann–Whitney U tests) shown in bold and comparisons that remain statistically significant after Bonferroni correction signalled with an asterisk.

Regarding information about the disease and written materials, Figure 2 shows that self-reported information was statistically significantly higher for patients not participating in clinical trials than for those who were.

**Figure 3:**
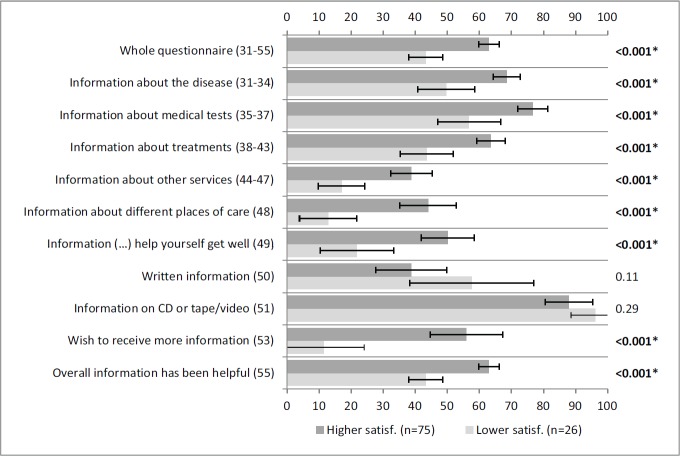
Self-reported information according to satisfaction with information. A lower satisfaction (lowest through mean) and higher satisfaction (mean through highest) correspond to answers to item 52 (‘satisfaction with the amount of information received‘) of INFO25. Vertical axis: INFO25 questions; horizontal axis: scores on the INFO25 questions (items range from 0 to 100). Higher scores mean a higher level of information received, higher information wishes, and higher satisfaction. Error bars represent 95% confidence intervals. *P*-values (Mann–Whitney *U* tests) appear on the right, with significant *p*-values shown in bold and comparisons that remain statistically significant after Bonferroni correction signalled with an asterisk.

**Figure 4:**
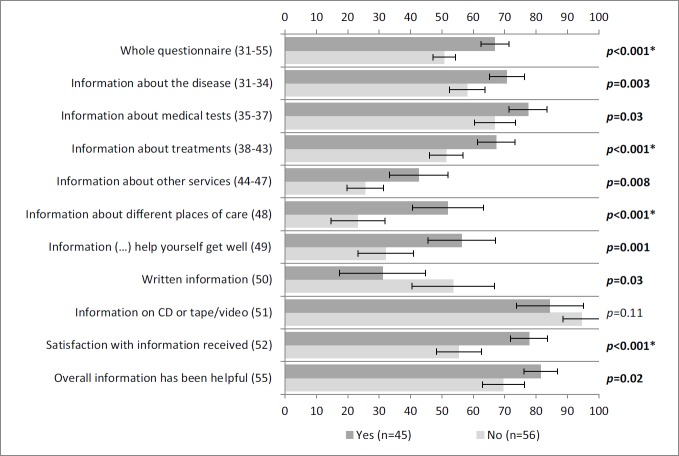
Self-reported information according to wishes for more information. More information wishes (yes) and less information wishes (no) correspond to answers to item 53a (‘Do you wish to receive more information?’) of INFO25. Vertical axis: INFO25 questions; horizontal axis: scores on the INFO25 questions (items range from 0 to 100). Higher scores mean a higher level of information received, higher information wishes, and higher satisfaction. Error bars represent 95% confidence intervals. *P*-values (Mann–Whitney *U* tests) appear on the right, with significant *p*-values shown in bold and comparisons that remain statistically significant after Bonferroni correction signalled with an asterisk.

Figure 3 portrays statistically significant associations between satisfaction with information and all the scales/items of INFO25 depicted, except for items 50 and 51. This means that patients were satisfied with the received information. Similar results were obtained for the association between information wishes and all the scales/items of INFO25 depicted, with the exception of item 51 (Figure 4).

## Discussion

Our study showed that patients attending the Day Hospital at IJB presented higher global information than the validation study sample [11], that their wishes to receive more information were lower and the helpfulness of the overall information received were higher [11].

Our study found similar data to the INFO25 validation study conducted by Arraras *et al *[11] regarding age distribution and gender. The education level of our sample was high, reflecting the educational attainment level of Belgium itself [18].

Open questions in health-related QoL evaluation questionnaires have been shown to be valued by patients, giving them freedom to list what they consider most important [19]; nevertheless, the analyses performed on these data are mainly qualitative. Patients wanting the results of medical tests stressed the desire to receive them on a hard-paper copy (e.g., blood tests or imaging), and also of having radiological findings explained to them during consultations, which has also been found by others [16]. Our working hypotheses *H1*, *H2*, and *H4 *to *H6 *were not supported. This is contrary to most published studies [1, 5, 6, 13, 15, 20, 21] and might be partially explained by the patients’ homogeneous and high levels of education.

Younger patients were more satisfied and found the information given more helpful than older patients (*H3*), which might derive from less attention so far given to the older cancer age groups regarding informational needs [22–24]. Nevertheless, older patients received more written and CD/tape/video information than younger patients. We can only speculate, but it might be an attempt of oncologists to overcome possible geriatric syndromes (e.g., hearing loss, cognitive dysfunction) in this age group by delivering information preferably on those formats [22].

Patients treated with palliative intent seem less well-informed about self-help and find the information given them less helpful. Others have shown that these patients have specific information needs, namely on physical symptoms, euthanasia [25], and complementary and alternative medicine (CAM) than patients treated with curative intent [13, 26] and that physicians are viewed as offering little useful input in this respect [20]. Furthermore, patients on palliative treatment or who received more than two lines of chemotherapy reported obtaining more written and CD/tape/video information than patients on earlier cancer stages, probably due to their longer disease duration, rendering them more likely to have received the results of computed tomography and positron emission tomography scans in such formats, which is becoming increasingly common.

Our exploratory analyses indicate that patients with higher information levels also report a higher QoL. More satisfied patients may require less additional information about specific topics, but they do continue to be open to novel information in general. However, our exploratory analyses indicated–unexpectedly–that patients outside clinical trials had more information about their disease, including more written information. Although we conducted additional statistical tests to look for confounding variables, none were found. These data mirror other recent evidence indicating that patients exhibit an unacceptably low understanding about the trials they join. Studies have shown that a fundamental factor in poor trial recruitment comes down to the communication skills of doctors [27, 28]. These are worrisome findings since they may be preventing many patients from the benefice of innovative therapies. On the other hand, interventions to improve the skills of doctors explaining clinical trials (Phase I, II, and III trials) do not suffice to provide key clinical information to patients [7, 27] and for them to understand it correctly. This underlines the urgent need to improve the content of patient informed sheet/consent forms, among other information provided to patients in connection with clinical trials.

We acknowledge that our study has some limitations, namely the fact that it was a single-centre study restricted to patients in an ambulatory setting. The sample contained both early and advanced cancer patients, preventing a clear separation among people in different disease stages and such, together with the fact that a longitudinal approach was not carried out, hinders the possibility of an assessment of the information given to cancer patients throughout the disease process from diagnosis to cure or end of life. It was also a single-institution study, and may therefore not represent the reality of Day Hospital patients’ of other institutions and also other countries.

Additional shortcomings concern INFO25 itself, particularly the items about the format in which information was given (written information and information on CD/tape/video). Unfortunately, those items do not discriminate which information is displayed in which support (it can refer to, e.g., test results, radiological images, and rehabilitation services announcements), possibly misleading patients in their responses, and thus rendering the interpretation of results challenging. Moreover, the item on CD/tape/video may even be considered outmoded, since information access presently emphasises the Internet and other technology-based health resources that are likely to rank high in terms of patients’ preferences, particularly the youngest [24, 28].

## Conclusions

Patients attending a day hospital in a busy comprehensive cancer centre in Europe are generally well-informed but would appreciate additional information on nutrition, long-term outcome, and recurrence symptoms, along with more details about medical tests and treatment side effects. The information perceived by patients in clinical trials is deficient, however, and interventional studies using a standardised format of delivering information are needed. INFO25 is a useful instrument for baseline assessment and intervention measures, although it would need to be updated to accommodate the role of new information sources available to patients, especially the Internet and other technology-based health tools that are now widespread.

## Conflicts of interest

The authors declare that they have no conflicts of interest.

## Figures and Tables

**Table 1.** Demographic and clinical characteristics of the patients.

Variable	*N*	%	Male	Female
Gender				
Male	22	21.8		
Female	79	78.2		
**Age**				
≤65 years		72	71.3	14
66–84 years	29	28.7	8	21
**Marital status**				
Single	15	14.9	2	13
Married	59	58.4	16	43
Divorced	18	17.8	4	14
Widow	6	5.9	0	6
Co-inhabitant	3	3.0	0	3
**Highest level of education**				
Primary	2	2	0	2
High school 9 years	9	8.9	2	7
High school 12 years	44	43.6	6	38
University degree	41	40.6	13	28
MSc/PhD	5	5.0	1	4
**Occupational sector**				
Business/Financial/Administrative	24	23.7	6	18
Education/Science	17	16.8	3	14
Trade/Other services	16	15.8	4	12
Health	12	11.9	1	11
Maintenance services	4	4.0	2	2
Marketing/Media	6	6.0	4	2
Protective services	1	1.0	0	1
Non-working	8	7.9	1	7
Not disclosed	13	12.9	1	12
**Place of birth**				
Belgium	75	74.3	16	59
Outside of Belgium	26	25.7	6	20
**Primary tumour site**				
Breast	59	58.4	0	59
Gastrointestinal	20	19.8	11	9
Urogenital	6	5.9	5	1
Gynaecological	5	5.0	0	5
Lung	5	5.0	3	2
Melanoma	3	3.0	2	1
Head and neck	2	2.0	1	1
Sarcoma	1	1.0	0	1
**Disease extent**				
Limited	35	34.7	5	30
Metastatic	66	65.3	17	49
**Performance status (ECOG)**				
0	29	28.7	7	22
1	59	58.4	11	48
2	13	12.9	4	9
**Line of treatment**				
Neo-adjuvant	11	10.9	2	9
Adjuvant	23	22.8	3	20
Metastatic				
• Approved (standard) treatments				
o 1st	20	19.8	6	14
o 2nd	10	9.9	4	6
o ≥3rd	16	15.9	1	15
• Non-approved treatments (clinical trial)	21	20.8	6	15
**Participation in clinical trial**^a^				
Yes	29	28.7	6	23
No	72	71.3	16	56

a Adjuvant and metastatic contexts.

**Table 2.** Descriptive statistics of INFO25 scales/items, reliability, and comparison with the module’s validation study.

	Present study		Arraras *et al* 2010					
Scales/items INFO25	*M ^a^*	*SD*	*M ^a^,^b^*	*SD^b^*	*p^c^*	Difference	*Mdn*	Cronbach’s alpha
Whole questionnaire (items 31–55)	57.9	16.4	43.6	13.4	< 0.001^*^	14.3	58.7	0.91
Information about the disease (items 31–34)	63.7	21.4	57.4	23.5	**0.01**	6.3	58.3	0.70
Information about medical tests (items 35–37)	71.6	23.7	67.7	26.9	0.18	3.9	66.7	0.83
Information about treatments (items 38–43)	58.4	21.8	48.7	20.7	< 0.001^*^	9.7	61.1	0.82
Information about other services (items 44–47)	33.2	28.2	29.4	22.3	0.14	3.8	25.0	0.75
Information about different places of care (item 48)	35.9	38.2	31.2	32.4	0.20	4.7	33.3	
Information about things you can do to help yourself get well (item 49)	42.9	36.9	39.3	34.5	0.35	3.6	33.3	
Written information (item 50)^d^	14.5	16.6	50.5	50.1	<0.001^*^	−36.0	0.0	
Information on CD or tape/video (item 51)^d^	30.0	10.0	5.4	22.6	< 0.001^*^	24.6	33.3	
Satisfaction with the information received (item 52)	65.3	26.6	63.7	29.1	0.61	1.6	66.7	
Wish to receive more information (item 53)^d^	14.9	16.7	47.7	50.1	< 0.001^*^	−32.8	0.0	
Overall the information has been helpful (item 55)	74.9	23.3	68.8	25.5	**0.03**	6.1	66.7	

*M* – Mean; *Mdn* – Median; *SD* – standard deviation.

a Scores in the INFO25 module scales and items range from 0 to 100. Higher scores mean a higher level of information received, higher information wishes, and higher satisfaction.

b Descriptive statistics from Arraras *et al *[11, Table 2], assessment during treatment (*n* = 451).

c Independent samples *t*-tests; significant *p*-values are shown in bold for ease of viewing.

d Items 50, 51, and 53 have a dichotomous answer (*yes*/*no*).

* Comparisons that remain statistically significant at a level of 0.05 after Bonferroni correction (the *p*-values reported are not corrected for multiple comparisons).
